# Vimentin is a dominant target of *in situ *humoral immunity in human lupus tubulointerstitial nephritis

**DOI:** 10.1186/ar4634

**Published:** 2014-09-18

**Authors:** Marcus R Clark, Anthony Chang, Kichul Ko, Carole J Henry Dunand, Scott Henderson, Dan Brandt, Natalya Kaverina, Vladimir Liarski, Patrick C Wilson, Andrew J Kinloch

**Affiliations:** 1 University of Chicago, Chicago, IL, USA

## Background

In lupus nephritis (LuN), severe tubulointerstitial inflammation (TII) predicts progression to renal failure. Severe TII is associated with tertiary lymphoid neogenesis and *in situ *antigen-driven clonal B-cell selection. The dominant autoantigen(s) driving *in situ *B-cell selection in TII are not known.

## Methods

Single CD38^+ ^or Ki-67^+ ^B cells were laser captured from seven LuN biopsies. Twenty clonally expanded immunoglobulin heavy and light chain variable region pairs were cloned and expressed as antibodies. Seven more antibodies were cloned from flow-sorted CD38^+ ^cells from an eighth biopsy. Antigen characterization was performed using a combination of confocal microscopy, ELISA, screening protoarrays, immunoprecipitation and mass spectrometry. Serum IgG titers to the dominant antigen were determined in 45 LuN and 38 non-nephritic lupus samples using purified antigen-coated arrays. Autoantigen expression was localized by immunohistochemistry and immunofluorescence on normal and LuN kidney.

## Results

Thirteen of 27 antibodies reacted with cytoplasmic structures, four reacted with nuclei and none with dsDNA, Sm or RNP. Vimentin was the only autoantigen identified by both mass spectrometry and on protoarray. Eleven anticytoplasmic TII antibodies directly bound vimentin. Vimentin was highly expressed by tubulointerstitial inflammatory cells, and tested TII antibodies preferentially bound inflamed tubulointerstitium. Finally, high titers of serum anti-vimentin antibodies were associated with severe TII (*P *= 0.0001).

## Conclusions

Vimentin, an antigenic feature of inflammation, is a dominant autoantigen targeted *in situ *in LuN TII. This adaptive autoimmune response probably feeds forward to worsen TII and renal damage.

